# Comparison of Four Methods for the *in vitro* Susceptibility Testing of Dermatophytes

**DOI:** 10.3389/fmicb.2020.01593

**Published:** 2020-07-14

**Authors:** Anthi-Marina Markantonatou, Konstantinos Samaras, Evaggelia Zachrou, Timoleon-Achilleas Vyzantiadis

**Affiliations:** First Department of Microbiology, Medical School, Aristotle University of Thessaloniki, Thessaloniki, Greece

**Keywords:** dermatophytes, susceptibility, EUCAST, resazurin, fragmented mycelia

## Abstract

**Objectives:**

Infections caused by dermatophytes affect a high percentage of the population. Antifungal susceptibility testing (AST) can offer useful information about the susceptibility profiles of the pathogens as well as the concomitant documentation of the appropriate treatment. However, the slow growth rate of these fungi and their poor sporulation are factors that can delay and affect the performance of the AST. The proposed methods by the CLSI or the EUCAST are both laborious for the everyday routine. There are alternative applications which propose the use of an inoculum, consisting of a conidia-mycelium mixture or even plain mycelia, as well as the use of resazurin in order to facilitate the reading. The aim of this study was to compare these approaches to the EUCAST method and evaluate their performance.

**Methods:**

Three alternative methods were compared to the EUCAST proposed methodology for conidia forming molds. The last was defined as the reference method. The methods under evaluation were (a) a fragmented mycelia method, (b) the EUCAST method with the addition of resazurin sodium salt solution and (c) the fragmented mycelia method with the addition of resazurin sodium salt solution. Twenty-two isolates (8 *Trichophyton interdigitale*, 8 *T. rubrum*, and 6 *Microsporum canis*) were tested against the antifungal agents of griseofulvin, terbinafine, fluconazole, and itraconazole.

**Results:**

The essential agreement between the methods was calculated in percentages and a statistical analysis of the results was performed. Data evaluation revealed sufficient overall agreement of the methods with the addition of resazurin to the initial “uncolored” methods (98.9 and 97.5% for the EUCAST and the fragmented mycelia methods, respectively). The fragmented mycelia method exhibited a relatively sufficient overall agreement in comparison to the EUCAST method (90%) and not a satisfactory correlation, probably as a result of various issues of standardization.

**Conclusion:**

The EUCAST method was found to be the more reliable one, whereas the addition of resazurin sodium salt solution facilitates the reading and provides a reliable and objective evaluation. The fragmented mycelia method could serve as an alternative that should be applied only in cases of poor or no sporulating dermatophytes.

## Introduction

Dermatophytoses are common fungal infections and they are transmitted quite easily, either between humans (anthropophilic species) or after contact with infected animals (zoophilic species). Although they are not life threatening like other fungal infections, they constitute a major problem for the affected individuals since they are often persistent, causing unpleasant symptoms that affect both physical and psychological health and significantly compromising the quality of life.

Their causative agents, the dermatophytes, are characterized by their ability to digest keratin. Thus they are considered primary pathogens that affect tissues rich in keratin, such as skin, nails and hair (Howell, 2018). The most common dermatophytic molds are *Trichophyton*, *Microsporum* and *Epidermophyton*. The new phylogeny of dermatophytes described by De Hoog et al. (2017) includes also the genera *Nannizzia*, *Lophophyton*, *Arthroderma*, and *Ctenomyces*, whereas two new genera- *Guarromyces* and *Paraphyton*- were introduced. Laboratory diagnosis of dermatophytoses is not always easy since there are certain issues that need to be addressed. The most important is that dermatophytic pathogens are mostly slow growing fungi and the time to release the final result of a culture is at least 2–3 weeks. Thus, the infection, at least initially, is usually treated by empirical administration of antifungal agents. The only antifungal drugs that the FDA of the United States has approved for the treatment of superficial mycoses are griseofulvin, terbinafine, ciclopirox and itraconazole (Del Rosso and Faocd, 2014) still other agents have demonstrated efficient *in vitro* action as well. Although empirical treatment seems to be effective in the majority of the patients, there are still some cases of persistent infections that do not respond to treatment or others that relapse. This may happen even due to poor compliance of the patients, since many superficial fungal infections demand long term therapies. For example, nail infections may need antifungal therapy from 3 to 12 months. Also, antifungal agents may demonstrate quite severe side effects, like hepatotoxicity. Besides, the administration of inappropriate drugs increases the cost of treatment. For all these reasons, the application of antifungal susceptibility testing (AST) could help to define the pathogens’ susceptibility patterns and probably contribute to the selection of the appropriate treatment with the more efficient and less toxic agent (Ghannoum et al., 2009).

Resistance to antifungal agents is an emerging problem that concerns pathogens mainly involved in invasive fungal diseases. However, it is possible to emerge in the case of dermatophytes as well (Mukherjee et al., 2003; Ghannoum et al., 2006; Saunte et al., 2019). High terbinafine resistance has been reported in *Trichophyton interdigitale* isolates in India (Singh et al., 2018), whereas Saunte et al. recently studied terbinafine-resistant *Trichophyton* isolates and detected already known, as well as novel mutations located at the squalene epoxidase target gene (Saunte et al., 2019). Furthermore, *in vitro* resistance has been correlated to treatment failure (Khurana et al., 2018). AST could be proved as a useful tool in order to monitor the potential development of resistance and could contribute toward its prevention, since the reveal of the appropriate antifungal agent, prevents from the unnecessary exposure to inappropriate agents.

The already mentioned slow growth rate of these fungi and their poor sporulation are factors that delay and may affect the performance of the susceptibility testing. Thus, up to now, there are not broadly used or commercialized and standardized techniques concerning AST in dermatophytes. Moreover, the proposed method by the CLSI (Clinical and Laboratory Standards Institute) (CLSI, 2008, 2010) or the modified applications of the EUCAST (European Committee on Antimicrobial Susceptibility Testing) method (Arendrup et al., 2017) are laborious for the everyday routine, resulting in the existence of only sparse information on the susceptibility patterns.

Acquired resistance patterns are not very common in dermatophytic isolates, and there are still only sparse reports of such cases (Mukherjee et al., 2003; Ghannoum et al., 2004, 2006). However, the application of antifungal susceptibility testing could serve multiple purposes, including effective therapeutic options, safe treatment with minimal toxicity, prevention of emerging resistance and decrease of the cost of treatment. The administration of inappropriate agents or in sub-therapeutic doses is possible to be one of the reasons of the emerging resistance to antifungal agents. The information acquired, concerning the susceptibility profile of the tested isolates, could be used to perform epidemiological studies, useful for the timely detection of potential emerging resistance. Still, antifungal susceptibility testing is not broadly applied yet, subsequently there are not sufficient laboratory and clinical data to support the aforementioned arguments. A wider application of AST concerning dermatophytic isolates in the future, in combination with clinical data could provide the information to prove the validity of these assertions.

Taking into consideration all the aforementioned facts, susceptibility testing of dermatophytes, could be encountered under a new perspective and even established in a broader scale. To achieve this, there is a need to restrict or even eliminate the two major issues that are responsible for the low frequency of AST application in dermatophytes, namely the technical difficulties and the lack of standardization. The aim of the present study was to compare the performance of four methods of antifungal susceptibility testing in dermatophytic isolates, to propose the easiest and more applicable one and try to determine the sensitivity profiles of the specific dermatophytic population tested. The four methods tested were: the EUCAST broth dilution method for moulds (Arendrup et al., 2017), a proposed method by the use of fragmented-mycelium inoculum, suitable for poor sporulating moulds (Schmalreck et al., 2012; Czaika and Schmalreck, 2014; Risslegger et al., 2015) and the alternative colorimetric versions of the previous methods by the use of resazurin sodium salt.

## Materials and Methods

### Isolates

All the dermatophytic isolates tested (8 *Trichophyton rubrum*, 6 *Microsporum canis*, and 8 *Trichophyton interdigitale* strains) belong to the collection of the First Department of Microbiology, Medical School, Aristotle University of Thessaloniki. They were clinical isolates that derived from patients coming from the geographical regions of Central (Thessally) and Northern (Macedonia and Thrace) Greece, suffering mainly from onychomycoses. The majority of the isolates was already stored in sterile water at 4°C and needed re-culture and a few of them were freshly harvested from the initial actual culture of the biological specimen. Stored isolates were at first cultured on Sabouraud Dextrose Agar with Chloramphenicol 0.05% -SDA (Laboratorios Conda S.A., Madrid, Spain) and then subcultured on Potato Dextrose Agar-PDA (Lab M Limited, Lancashire, United Kingdom) in order to enhance sporulation.

### Identification

The identification of the isolates was performed phenotypically by standard mycological techniques, based on their macroscopic and microscopic features as well as on their biochemical and pathophysiological properties (urease production and ability for hair penetration).

### Methods

#### The Reference Method

EUCAST method (version 9.3.1) for the determination of broth dilution minimum inhibitory concentrations of antifungal agents for conidia forming moulds was defined as the reference method of this study (Arendrup et al., 2017). It was performed in flat-bottom microdilution plates (96 well Cell Culture Cluster, Flat Bottom with Lid, Tissue Culture Treated, Non-Pyrogenic, Polystyrene, Costar^®^, Corning Incorporated, New York), as recently proposed (Arendrup et al., 2017). RPMI 1640 with L-glutamine, without glucose and NaHCO_3_ (Sigma-Aldrich^TM^) supplemented with glucose to a final concentration of 2% and buffered with MOPS [3-(N-Morpholino)propanesulfonic acid] (AppliChem GmbH, Darmstadt, Germany) was the culture medium, as recommended. A standardized inoculum was prepared accordingly.

Using a damp sterile cotton swab, conidia from fresh mature (adequate conidiogenesis was evident at least after 2 weeks of incubation) cultures on PDA (usually from 2 to 3 Petri dishes of 6 cm) were carefully rubbed and transferred into a sterile tube containing 5 ml of sterile water, supplemented with 0.1% Tween 20. The suspension was vortexed for 15 s and further diluted in order to attain an appropriate concentration in order to be counted by the means of a haemocytometer. Usually a dilution of 1:10 was adequate. The preparation was examined for the presence of hyphae or clumps. If they were present in a percentage of more than 5%, the inoculum was filtrated through eight layers of sterile gauze, so as to obtain a suspension composed mainly from conidia. After the counting of the conidia the inoculum was further diluted accordingly in order to obtain the recommended final concentration of 2–5 × 10^5^ conidia/mL, which is equal to 2–5 × 10^5^ CFU/mL. Subsequently 100 μL of the inoculum was added to every well of the plate except for the last column (12), where only sterile water was added to the culture medium, as it was predefined to represent the negative control. Wells in column 11 were containing culture medium (RPMI/MOPS) without any antifungal drug, and were used as a growth control. After the inoculation of the plates, a small proportion of the suspension was diluted (1:100) and inoculated in SDA in order to verify the number of present viable units, expressed as Colony Forming Units-CFU/mL. After an incubation period of 5 days in 35°C, the endpoint was read visually, comparing the degree of growth at each well in comparison to growth control wells, and minimum inhibitory concentration (MIC) was determined. Appropriate control strains (*Aspergillus fumigatus* ATCC 204305 and *Candida albicans* ATCC 90028) were used to ensure the quality of the results.

#### The Methods Under Evaluation

(A) The modified method of fragmented-mycelium inoculum for poor sporulating molds (Schmalreck et al., 2012; Czaika and Schmalreck, 2014; Risslegger et al., 2015). The isolates were subcultured on PDA. As soon as adequate mycelium development occurred, sterile water plus Tween 20 (in a proportion of 0.1%) was poured onto the colonies and they were gently probed with a sterile cotton swab. Usually 1–2 petri dishes (diameter of 6 cm) and 5 mL of sterile water were enough. The probes of mycelia and conidia were transferred into safe-lock microcentrifuge tubes (SafeSeal tube 1.5 mL, Sarstedt, Nümbrecht, Germany), containing acid-washed glass beads (Sigma-Aldrich^TM^), and homogenized for at least 15 min, by the means of a Turbo Mix (Vortex-Genic 2, Scientific Industries, Inc.). The suspension was examined microscopically, and if aggregations or clumps of hyphae were present, further homogenization was applied. Consequently, the suspension was diluted in order to be countable by the means of a haemocytometer. After counting, further dilution was applied in order to obtain an inoculum of 2–5 × 10^4^ viable units (VU)/ml. Viable Units were defined as the viable and uniform particles in a homogenous suspension, represented either by conidia or by hyphal segments framed by intact septa. The suspension was inoculated on the plates (96 well Cell Culture Cluster, Flat Bottom with Lid, Tissue Culture Treated, Non-Pyrogenic, Polystyrene, Costar, Corning Incorporated, New York) by the use of the same pattern as in the EUCAST method. Finally, the inoculum was checked by plating 100 μl (diluted 1:10) onto Sabouraud agar plates. The accuracy of the inoculum was verified by the comparison of the number of colony forming units (CFU) and VU counted in the haemocytometer, respectively. MIC endpoint determinations were performed visually after an incubation period of 5 to 11 days at 35°C.

(B) The two alternative colorimetric methods by the use of resazurin sodium salt (Sigma-Aldrich). Resazurin solution was used as a growth indicator and it was prepared as follows: resazurin sodium salt powder was weighted in a recently calibrated precision scale and subsequently diluted in phosphate-buffered saline, PBS (Invitrogen Corporation) in order to prepare a stock solution with a 10-fold concentration of the finally required. PBS and the stock solution as well, were filter sterilized through filters with a pore size of 0.2 μm (Filtropur S 0.20 μm, Sarstedt). Stock solution was diluted right before use, in a final concentration of 440 μM as previously proposed (O’Brien et al., 2000). The principle was applied twice in two different types of inoculum. The first one was prepared as described in the aforementioned fragmented mycelia method and the other one according to the EUCAST method guidelines. In comparison to the two previous methods (EUCAST broth dilution and modified “fragmented-mycelium inoculum” method), the only difference is the addition of the resazurin sodium salt solution at the RPMI/MOPS medium in a proportion of 10% v/v. The MIC determinations were performed visually. The reading of the plates was facilitated by the fact that there was a color change, from blue to pink, when there were viable cells in the solution.

In all four methods the four most commonly administrated antifungal agents for dermatophytoses, namely fluconazole (fluconazole 98.5%, J&K Scientific GmbH, Pforzheim, Germany), itraconazole (itraconazole 99.5%, Janssen Pharmaceutica N.V., Beerse, Belgium), terbinafine (terbinafine hydrochloride 98%, J&K Scientific GmbH, Pforzheim, Germany) and griseofulvin (griseofulvin 99.6%, J&K Scientific GmbH, Pforzheim, Germany) were tested. A 2-fold dilution series were prepared in the RPMI/MOPS culture medium and the drug concentrations ranged from 0.25 to 128 mg/L for fluconazole and from 0.031 to 16 mg/L for itraconazole, terbinafine and griseofulvin.

#### Drug Preparation

Every powder drug was weighted in a recently calibrated precision scale and subsequently diluted in DMSO [Dimethylsulfoxid (CH3)_2_SO, Merck Schuchardt, München, Germany] in order to obtain stock solutions with 100-fold concentrations of the highest finally required. Stock solutions were prepared in microcentrifuge tubes and stored at −20°C until used.

#### Plate Preparation

For every drug serial 2-fold dilutions were prepared in the RPMI/MOPS culture medium from stock solutions after a 1:100 initial dilution. In every row, columns 1–10, were containing 100 μL of serial dilutions and columns 11 and 12 drug free medium. Column 11 was inoculated with fungal suspension and used as a growth control whereas in column 12, only solvent (sterile water) was added and it was used as a negative control. Each 96 well plate (12 × 8) was used for the testing of only one isolate. The first four rows were used to apply either the EUCAST or the fragmented mycelia method, whereas the last four rows were used to apply the same method with the addition of resazurin solution.

#### Incubation

The plates were incubated at 35°C for 7 days and they were read visually at days 3, 5, and 7.

#### Endpoint Determination and Evaluation of the MIC

The endpoint determination was conducted at day 5 for the EUCAST method and as soon as adequate growth was achieved at the growth control well for the fragmented mycelia method. The MICs for terbinafine and griseofulvin were determined as the concentration that achieved complete inhibition of the fungal growth whereas for itraconazole and fluconazole as the concentration that achieved an inhibition of at least 80%.

#### Analysis of Results

The fragmented mycelia method and the EUCAST method with the addition of resazurin were compared to the EUCAST method which was defined as the reference method. Also the fragmented mycelia method with the addition of resazurin was compared to the fragmented mycelia method. Analysis was conducted for the results obtained after 5 and 7 days of incubation. In few cases the fragmented-mycelia method required more than 7 days to demonstrate efficient growth in the growth control wells, thus the endpoint determination was defined as soon as efficient growth was obtained. Both on-scale and off-scale results were included in the analysis. The obtained low off-scale results were left unchanged. The comparisons were conducted mainly by the definition of essential agreement, which included discrepancies of no more than ±2 serial 2-fold dilutions. The statistical analysis of the results included *t*-test after a log_2_ transformation of the MICs as well as a correlation study (Pearson’s *r*) between each couple of methods under evaluation. The results obtained by the EUCAST method and by the fragmented mycelia method after 5 and 7 days of incubation respectively, were tested by paired samples *t*-test. The statistical analysis was conducted by the means of the Statistical Package of the Social Sciences software (IBM SPSS Statistics, version 20). *P* values equal or lower than 0.05 were considered statistically significant. Half of the isolates of the study were tested at least two times in an effort to test the reproducibility of double measurements.

## Results

**FIGURE 1:**
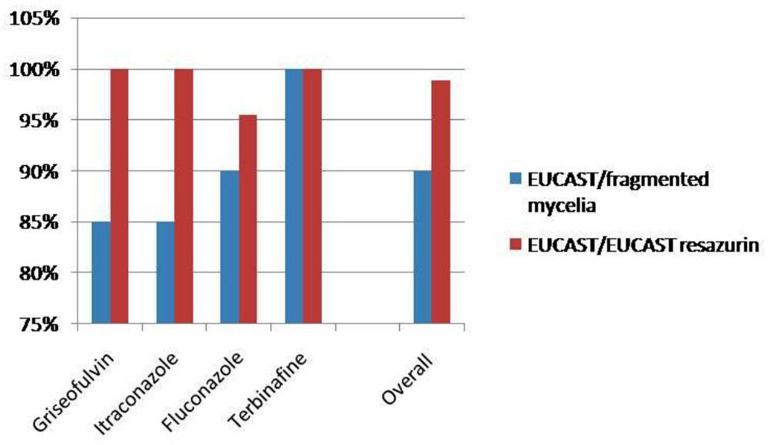
Comparison of the essential agreement between EUCAST and fragmented mycelia method with the agreement between EUCAST and EUCAST with the addition of resazurin.

The MICs obtained by the application of EUCAST and fragmented mycelia method are demonstrated in Table 1 whereas Table 2 demonstrates the MICs distribution as concerns the EUCAST method results. MIC ranges and MIC_50_ (MIC at which the 50% of the isolates are inhibited) defined by the EUCAST method are demonstrated in Table 3. A graphic depiction that compares the essential agreement between EUCAST and fragmented mycelia method with the agreement between EUCAST and EUCAST with the addition of resazurin is demonstrated in Figure 1.

**TABLE 1:** MICs obtained by the EUCAST and the fragmented mycelia methods (mg/L).

	EUCAST method				Fragmented mycelia method			
	griseo	itra	fluco	terbi	griseo	itra	fluco	terbi
***T. rubrum***								
1	2	0.25	4	<0.031	1	0.125	2	<0.031
2	4	1	8	<0.031	2	0.5	4	<0.031
3	4	2	32	<0.031	2	0.5	8	<0.031
4	4	1	16	<0.031	0.5	0.125	4	<0.031
5	4	2	64	0.063	0.5	0.125	4	<0.031
6	1	0.25	32	<0.031	2	0.5	64	<0.031
7	2	0.5	32	<0.031	2	0.5	16	<0.031
8	1	0.25	8	<0.031	1	0.125	4	<0.031
***T. interdigitale***								
1	1	0.25	4	<0.031	0.5	0.5	4	<0.031
2	1	0.125	32	<0.031	1	0.125	32	<0.031
3	1	0.25	8	<0.031	1	0.125	2	<0.031
4	4	0.25	64	<0.031	1	0.25	64	<0.031
5	2	0.5	8	<0.031	2	0.25	32	<0.031
6	1	0.5	128	<0.031	0.031	0.063	8	<0.031
7	1	0.063	8	<0.031	0.5	0.125	16	<0.031
8	2	1	16	<0.031	2	0.25	4	<0.031
***M. canis***								
1	0.25	0.125	8	<0.031	0.5	0.125	8	<0.031
2	1	1	16	0.063	0.5	0.25	8	<0.031
3	0.5	0.125	16	<0.031	0.25	0.031	4	<0.031
4	1	0.5	16	<0.031	1	0.25	8	<0.031
5	0.25	0.125	16	<0.031	*	*	*	*
6	0.5	0.125	8	<0.031	*	*	*	*

*The fragmented mycelia method didn’t produce results in those cases. Griseo, griseofulvin; itra, itraconazole; fluco, fluconazole; terbi, terbinafine.

**TABLE 2:** Minimum inhibitory concentrations (MIC) distribution of griseofulvin, itraconazole, fluconazole, and terbinafine against the isolates tested by the EUCAST broth microdilution method (mg/L).

MICs	<0.031	0.063	0.125	0.25	0.5	1	2	4	8	16	32	64	128
***T. interdigitale*** (8)													
Griseofulvin						5	2	1					
Itraconazole		1	1	3	2	1							
Fluconazole								1	3	1	1	1	1
Terbinafine	8												
***T. rubrum*** (8)													
Griseofulvin						2	2	4					
Itraconazole				3	1	2	2						
Fluconazole								1	2	1	3	1	
Terbinafine	7	1											
***M. canis*** (6)													
Griseofulvin				2	2	2							
Itraconazole			4		1	1							
Fluconazole									2	4			
Terbinafine	5	1											
**Total distribution**													
Griseofulvin				2	2	9	4	5					
Itraconazole		1	5	6	4	4	2						
Fluconazole								2	7	6	4	2	1
Terbinafine	20	2											

**TABLE 3:** Minimum inhibitory concentration (MIC) ranges and MIC_50_ of griseofulvin, itraconazole, fluconazole and terbinafine against the isolates tested by EUCAST broth microdilution method (mg/L).

	MIC range	MIC_50_
***T. interdigitale* (8)**		
Griseofulvin	1–4	1
Itraconazole	0.063–1	0.25
Fluconazole	4–128	8
Terbinafine	<0.031–<0.031	<0.031
***T. rubrum* (8)**		
Griseofulvin	1–4	2
Itraconazole	0.25–2	0.5
Fluconazole	4–64	16
Terbinafine	<0.031–0.063	<0.031
***M. canis* (6)**		
Griseofulvin	0.25–1	0.5
Itraconazole	0.125–1	0.125
Fluconazole	8–16	16
Terbinafine	<0.031–0.063	<0.031
**All the isolates (22)**		
Griseofulvin	0.25–4	1
Itraconazole	0.063–2	0.25
Fluconazole	4–128	16
Terbinafine	<0.031–0.063	<0.031

### Data Analysis

#### After 3 Days of Incubation

The vast majority of the isolates tested exhibited either no growth or a minimal growth but not adequate to allow determination of the MICs.

#### After 5 Days of Incubation

All the isolates tested with the EUCAST reference method, as well as with the addition of resazurin solution, exhibited sufficient growth to allow plate reading and definition of the MICs. A significant percentage of the isolates tested by the fragmented mycelia method, with or without resazurin solution, exhibited a sufficient growth too. Isolates that were not ready to be evaluated in the fifth day were excluded from the comparisons at this point and were further incubated.

Neither of the *M. canis* isolates exhibited efficient growth at day 5, as concerns the fragmented mycelia method with or without resazurin.

#### After 7 Days of Incubation

The majority of the isolates exhibited adequate growth to define MICs except for five isolates tested by the fragmented mycelia method, which were excluded from the comparisons at this point and again were left for further incubation.

#### Comparison Between 5 and 7 Days of Incubation

The duration of incubation was evaluated for the EUCAST reference method as well as for the fragmented mycelia method. It was observed that longer periods of incubation yielded higher MICs. As concerns the EUCAST method there was a statistically significant difference for the MICs of itraconazole and fluconazole (*p* = 0.004 and 0.048, respectively), whereas the difference was not significant for griseofulvin and terbinafine. As for the fragmented mycelia method, there was a statistically significant difference for the MICs of griseofulvin, itraconazole and fluconazole (*p* = 0.039, 0.005, and 0.009, respectively).

#### Comparisons Based on the Endpoint Measurements

As shown above, there were some isolates that did not exhibit sufficient growth at day 7 and they were excluded from the calculations at that point. In order to obtain a total aspect of the susceptibility profiles of the strains tested and to perform the required comparisons, the following convention was accepted: As concerns the EUCAST method and the EUCAST method with the addition of resazurin, the MIC was defined after 5 days of incubation. As concerns the fragmented mycelia method, with or without resazurin solution, the MIC was defined once adequate growth was exhibited at the growth control wells.

According to the above described condition the fragmented mycelia method compared to EUCAST demonstrated an essential agreement of 90% whereas the addition of resazurin in EUCAST demonstrated an essential agreement of 98.9% with the EUCAST method. The addition of resazurin in fragmented mycelia method demonstrated an essential agreement of 97.5% with the fragmented mycelia method without resazurin (Table 4).

**TABLE 4:** Essential agreement between the methods under evaluation.

	EUCAST/fragmented mycelia	EUCAST/EUCAST-resazurin	Fragmented mycelia/fragmented mycelia-resazurin
***T. interdigitale*** (8)			
Griseofulvin	7/8 (87.5%)	8/8 (100%)	7/8 (87.5%)
Itraconazole	7/8 (87.5%)	8/8 (100%)	8/8 (100%)
Fluconazole	7/8 (87.5%)	7/8 (87.5%)	8/8 (100%)
Terbinafine	8/8 (100%)	8/8 (100%)	8/8 (100%)
***T. rubrum*** (8)			
Griseofulvin	6/8 (75%)	8/8 (100%)	8/8 (100%)
Itraconazole	6/8 (75%)	8/8 (100%)	8/8 (100%)
Fluconazole	7/8 (87.5%)	8/8 (100%)	8/8 (100%)
Terbinafine	8/8 (100%)	8/8 (100%)	8/8 (100%)
***M. canis*** (6)*			
Griseofulvin	4/4 (100%)	6/6 (100%)	4/4 (100%)
Itraconazole	4/4 (100%)	6/6 (100%)	4/4 (100%)
Fluconazole	4/4 (100%)	6/6 (100%)	3/4 (75%)
Terbinafine	4/4 (100%)	6/6 (100%)	4/4 (100%)
**All the isolates** (22)*			
Griseofulvin	17/20 (85%)	22/22 (100%)	19/20 (95%)
Itraconazole	17/20 (85%)	22/22 (100%)	20/20 (100%)
Fluconazole	18/20 (90%)	21/22 (95.5%)	19/20 (95%)
Terbinafine	20/20 (100%)	22/22 (100%)	20/20 (100%)
**Overall**	72/80 (90%)	87/88 (98.9%)	78/80 (97.5%)

*^∗^The fragmented mycelia method didn’t produce results in two cases.*

The overall comparison of the fragmented mycelia method to the EUCAST method, exhibited a statistically significant difference for itraconazole and fluconazole (*p* = 0.038 and 0.035, respectively). When each fungus was examined separately, statistically significant differences were observed for griseofulvin as concerns *T. rubrum* isolates (*p* = 0.041) and fluconazole as concerns *M. canis* isolates (*p* = 0.024). Additionally, correlation studies demonstrated that there was no significant correlation between these two methods, either in the total population or separately in each isolate.

The comparison of the EUCAST method with the addition of resazurin to the reference method, exhibited that there were not significant differences for any of the drugs, either totally or when each fungus was examined separately (*p* values from 0.114 to 1). Correlation studies demonstrated satisfactory results in the total population for all the antifungal agents (griseofulvin: *r* = 0.796, *p* < 0.001, itraconazole: *r* = 0.856, *p* < 0.001, fluconazole: *r* = 0.625, *p* = 0.002, terbinafine *r* = 0.690, *p* < 0.001) and similarly for each fungus separately, with the exception of griseofulvin for *T. interdigitale* (*r* = 0.267, *p* = 0.522), and *M. canis* (*r* = 0.612, *p* = 0.196) and fluconazole for *M. canis* (*r* = 0.463, *p* = 0.355).

The comparison of the fragmented mycelia method with or without the addition of resazurin exhibited not significant differences for any of the antifungals, either totally or for each fungus separately (*p* values from 0.144 to 1). Correlation studies demonstrated good correlation (*r*: from 0.549 to 1.0, *p*: from <0.001 to 0.013) in the total population for all the agents tested, as well as for each fungus separately, with the exception of griseofulvin for *T. interdigitale* (*r* = 0.552, *p* = 0.156), *T. rubrum* (*r* = 0.234, *p* = 0.578) and *M. canis* (*r* = 0.866, *p* = 0.134) and itraconazole for *T. interdigitale* (*r* = 0.652, *p* = 0.080) and *M. canis* (*r* = 0.667, *p* = 0.333).

#### Reproducibility of Double Measurements

The convention made to evaluate reproducibility was the same as the one made for the evaluation of essential agreement. Discrepancies of no more than ±2 serial 2-fold dilutions were accepted as reproduction of the result. Analysis of the results obtained from double measurements, conducted for each one of the evaluated methods and demonstrated excellent reproducibility with minimal discrepancies in only ten cases, where the difference concerned 3–4 serial sub-dilutions.

## Discussion

Dermatophytes are quite easily transmitted primary pathogens that affect a significant percentage of the population. Although they rarely cause severe infections, they may significantly affect physical and psychological health of the patients. This kind of infections is usually treated with the administration of empirical antifungal therapy. However, the efficacy of the treatment requires a few weeks to be revealed and frequently there are cases that do not respond to treatment or concern relapsing infections. Resistance to antifungals (although not common) has been observed (Mukherjee et al., 2003; Ghannoum et al., 2004, 2006).

Antifungal susceptibility testing (AST) of the pathogens is a useful tool to define the susceptibility profile, to monitor resistance and also a means to prevent it. It can contribute to choose the appropriate antifungal agent that combines the better efficacy with the less toxicity. Bearing in mind that antifungal agents are often quite expensive drugs, AST could also help to reduce the cost of treatment.

The major issue regarding antifungal susceptibility testing in dermatophytes is that of standardization, which is necessary in order to obtain comparable results from different laboratories. Efforts for standardization have been done from investigators, who tried to optimize the individual components of the procedure, such as culture medium, inoculum size, growth conditions and the endpoint determination (Norris et al., 1999; Jessup et al., 2000).

Technical reasons for not applying AST in dermatophytes are their slow rate of growth and low conidiogenesis. Modifications of the already existing methods have been proposed to overlap these issues (Schmalreck et al., 2012; Czaika and Schmalreck, 2014; Risslegger et al., 2015). In the current study an effort was made to evaluate these modifications as well as alternative colorimetric methods that could facilitate the MIC determination.

The results of the experiments were used in order to conduct a comparative study that included: (a) Comparison of the fragmented mycelia method to the EUCAST method which was defined as the reference method for this experiment. (b) Evaluation of the probable facilitation offered by the addition of resazurin solution or not at the two previous methods. (c) Summarizing the results of the AST and definition of MIC ranges and MIC_50_ for the species included in the study.

Several issues occurred during the process of the study that led to some interesting and useful conclusion.

**FIGURE 2:**
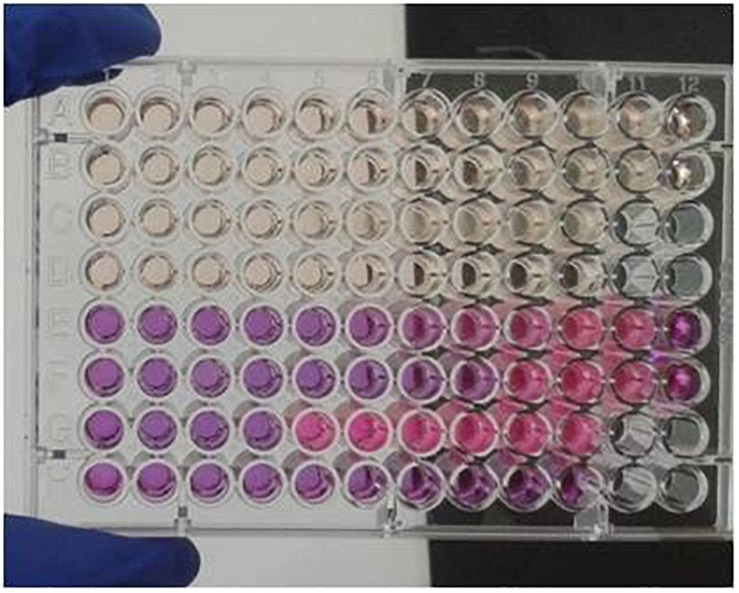
View of a plate at day 5. In every row, columns 1–10, contain serial dilutions of the antifungals. Column 11 includes growth controls and column 12 negative controls. Rows E, F, G and H contain resazurin sodium salt solution. Antifungal agents: rows A,E: griseofulvin, B,F: itraconazole, C,G: fluconazole, D,H: terbinafine.

**FIGURE 3:**
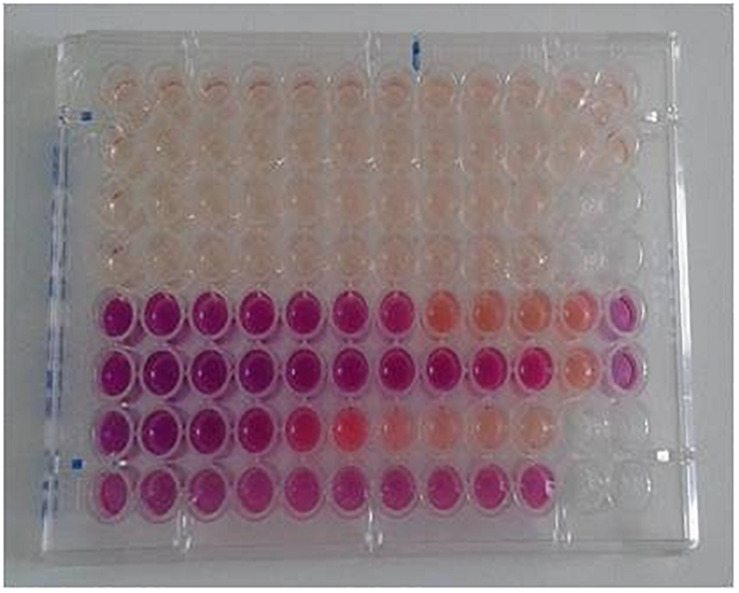
View of a plate after 10 days of incubation. The over-reduction of resazurin has yielded the uncolored product hydroresorufin.

Resazurin sodium salt solution was used in this study as a growth indicator. Resazurin is a chemical substance of blue color, which can be reduced to pink resorufin, and further reduced to uncolored hydroresorufin. The “resazurin reduction test” has been used to identify the presence of pathogens in milk or to test the viability of cells or microorganisms in various experiments, since the presence of live organisms induces the reduction of the substance (O’Brien et al., 2000). O’Brien et al. (2000) proved the identity of Alamar blue as resazurin. By the use of a resazurin standard curve, they defined the resazurin concentration of the commercial Alamar Blue solution at 440 μM. Taking into consideration the aforementioned information, a resazurin solution of 440 μM was used in our experiments to test the viability of the fungus in each well. Figures 2, 3 demonstrate the appearance of the plates at the time of endpoint determination and after several days of incubation, respectively.

During the preliminary experiments there was an observation that resazurin solution was reduced very easily, even before the addition of the fungal inoculum. An effort was made, to keep already prepared plates, loaded with diluted drugs as well as with the colored solution, at −20°C. The results were disappointing, since resazurin was already reduced when the plates were took out of the freezer. Similar results were obtained even if the colored solution remained in the plates for 2–3 h before loading the inoculum. Thus, the decision made was to add the resazurin solution in the wells right before the fungal suspension. Once that practice was applied, the results were excellent, since there was a great facilitation regarding the reading of the plates and the results were comparable to these obtained with each one of the “original uncolored” methods.

For many years there were no official guidelines as concerns AST in dermatophytes. In 2008 CLSI included dermatophytic isolates in the guidelines regarding AST of filamentous fungi and further modifications were proposed in 2010 (CLSI, 2008, 2010). Nevertheless, there are still difficulties concerning the application of the procedure. Ordinarily, an inoculum consisting of a specific number of conidia is required to perform the test. Conidia provide the advantages of easy counting and concrete definition of the viable units’ number, and thus facilitate standardization of the method. The most important issue is dermatophytes’ slow rate of growth as well as poor sporulation of individual species (e.g., *Trichophyton rubrum*). As a result a long period of time is required in order to obtain efficient sporulation and perform the AST.

In an effort to provide an alternative, some investigators have proposed the use of an inoculum consisting of a conidia-mycelium mixture or even from plain mycelia. First, Granade and Artis (1980), managed to prepare an inoculum free of conidia, consisting of plain fragmented mycelia. They defined that the optimum inoculum should have an absorbance of 0.600 at 450 nm. Schmalreck et al. (2012) evaluated an inoculum consisting of a mixture of conidia, hyphae and vegetative cells, in non-dermatophytic moulds, whereas Czaika et al. applied this type of inoculum in AST of dermatophytic isolates (Czaika and Schmalreck, 2014). Risslegger et al. (2015) compared the aforementioned method to CLSI (2008) and EUCAST (Subcommittee on Antifungal Susceptibility Testing of the Escmid European Committee for Antimicrobial Susceptibility Testing, 2008) methods and concluded that it is highly comparable to them. Although a conidia-mycelium mixture inoculum is easy to prepare, there is always the important issue of standardization since the number of viable units is not possible to be exactly known in advance.

As concerns the preparation of the “fragmented mycelia” inoculum, there were some significant issues. The investigators that had previously described the method, had applied fragmentation into IKA-BMT-20 (IKA, Germany) mixing vessels, containing stainless steel balls by the use of the IKA-Ultra-Turrax TubeDrive homogenization tool (IKA, Germany) (Schmalreck et al., 2012; Czaika and Schmalreck, 2014; Risslegger et al., 2015). Since the specific tools were not available in our laboratory, the decision was to apply some modifications in the fragmentation method. Thus, instead of the aforementioned tools, safe-lock microcentrifuge tubes, containing glass beads, were used. The homogenization was performed by vigorous agitation in the Turbo mix tool for at least 15 min. In many cases a quite homogenous suspension was obtained whereas in other cases aggregations or clumps of hyphae occurred. Once that happened, further agitation was applied, however the aggregations were not always dissolved. In most of these cases the susceptibility testing failed to be performed or yielded inconsistent results.

Since the fungal solution prepared with this method is not always absolutely homogenous, an issue of standardization was revealed. Additionally, in many cases, considerable numbers of conidia were present, resulting in a mixed solution. The observation made, was that the highest percentage of conidia present, the sooner the plates were ready for reading and endpoint determination. Thus, a second issue of standardization occurred concerning the necessary incubation time for the determination of MICs. In other cases, independently of the presence or not of conidia, there was a great variability regarding the duration of incubation required until an adequate growth was exhibited in growth control wells. As previously observed there was a significant delay in growth, due to the slower proliferation of hyphae in comparison to conidia (Risslegger et al., 2015). There were isolates that needed up to 11 days to produce sufficient growth.

Some investigators have expressed arguments to support the superiority of the fragmented mycelia inoculum over fungal solutions consisting only of conidia, whereas others emphasize the advantages of conidial inocula. Czaika and Schmalreck present as disadvantages of the latter the facts of not consistent presence, difficult harvesting and purifying and their slow formation in the cultures (Czaika and Schmalreck, 2014). On the other hand, they support that mycelium elements grow much faster and can easily disperse and form homogenous solutions of viable units easy to be counted. Additionally, they present as an advantage the fact that the mycelia are closer to the hyphal forms found in the infected individuals and present lower variation in comparison to conidia.

However, the method is not officially recommended by CLSI or EUCAST (CLSI, 2008, 2010; Arendrup et al., 2017) since conidial suspensions are much easier to be standardized. Also, various methods used to produce fragmented mycelia inocula were either laborious or expensive from financial and logistical aspect (Granade and Artis, 1980; Burden, 2008). Another issue occurring is that of the high percentage of non-viable cell fragments (Burden, 2008).

Statistical analysis in this study demonstrated not significant correlation of the method in comparison to EUCAST reference method. An interesting observation was that in the fragmented mycelia method shortest time was required to obtain the necessary mycelium to prepare the fungal inoculum, but longer and not clearly defined duration of incubation was required to evaluate the test. Conversely, in the EUCAST reference method, longer time was required to obtain sufficient sporulation to prepare the conidial inoculum, but shortest and well defined duration of incubation was efficient to estimate the MICs. Nevertheless, the results of the fragmented mycelia method were comparable to these obtained by the reference method. Subsequently, it could be a suitable approach, in cases of poor or no sporulating molds serving as an alternative method. In case that adequate sporulation could be obtained in a sufficient short time interval, the EUCAST method should be preferred.

In order to define the optimal incubation time for the determination of MICs, the plates were evaluated at three “snapshots”, namely days 3, 5, and 7. Most of the isolates did not exhibit any growth at day 3 or they exhibited a minimal growth, not efficient to conduct plate reading. As regards day 5, all isolates tested with the EUCAST reference method as well as the same method with the addition of resazurin solution could be reliably studied. On the other hand, a significant percentage of the isolates tested with the fragmented mycelia method did not exhibit sufficient growth at day 5 and some of them not even at day 7 or 9. This fact led to the convention of reading the EUCAST method with or without the addition of resazurin solution at day 5, whereas the fragmented mycelia method with or without the addition of resazurin solution, as soon as an adequate growth was exhibited at the growth control wells.

The antifungal agents tested in this study were griseofulvin, itraconazole, fluconazole and terbinafine. Griseofulvin is a fungistatic drug that inhibits fungal cell mitosis by disorientating microtubules. Itraconazole and fluconazole, are mainly fungistatic antifungal agents that inhibit 14α-sterol demethylase and thus induce alterations of membrane fluidity. On the other hand, the allylamine antifungal terbinafine is an agent that inhibits squalene epoxidase of the ergosterol pathway, and thus acts as a fungicidal drug *in vitro* (Ghannoum and Rice, 1999).

Determination of the endpoint is a quite subjective issue and it is one of the sources of interlaboratory discordance. The optimal approach is to estimate a prominent decrease in turbidity in comparison to the control well. Although EUCAST does not provide endpoints for dermatophytes, CLSI propose an 80% or more reduction in growth, in comparison to the growth control well (CLSI, 2008, 2010). Klepser et al. (1998) propose that an 80 and a 100% inhibition should be considered for fungistatic and fungicidal drugs, respectively.

The convention made for the endpoint determination in this work, was to evaluate absolute inhibition of growth for terbinafine and griseofulvin, whereas for itraconazole and fluconazole an 80% inhibition of growth, in comparison to growth control.

Following the aforementioned convention, MICs for each one of the antifungals were defined for all the isolates by the means of the four methods under evaluation. In general, the distribution of MIC results obtained by the application of a specific method is used to define the wild-type distribution. The latter is used to define epidemiological cut-off (ECOFF) concentrations which are used as indicators of the activity of the drug (Johnson, 2018). The values at which 50 and 90% of the isolates are inhibited are defined as MIC_50_ and MIC_90_, respectively. When clinical data are available, there is the possibility to define clinical breakpoints by the combination of information related to the normal MIC ranges of the fungus, the expected levels of the drug in infected tissues and the relevant clinical outcome. Since infections caused by dermatophytes are not life threatening, there is a lack of extended clinical studies to provide efficient data in order to establish clinical breakpoints. Thus, only epidemiological cut-offs could be defined, however a large number of isolates is required. Although this study concerned a finite limited number of isolates an attempt to define MIC_50_ was done.

The current study was quite novel since dermatophytes are not extensively studied concerning their sensitivity profiles. There was an effort to introduce methods that facilitate antifungal susceptibility testing, in terms of prompt release of the result (fragmented mycelia method) and more objective evaluation of the endpoint (addition of resazurin solution). The study was conducted under strict predefined terms and the results were utilized to exclude useful conclusions. However, it concerned only an intralaboratory study. A larger amount of isolates in combined interlaboratory studies could further confirm the conclusions of this study and moderate any subjective factor.

Summarizing the results of this study, the EUCAST reference method is the most reliable method to apply for the antifungal susceptibility testing of dermatophytes. The application of the method with the addition of resazurin sodium salt solution facilitates reading whereas renders reliable results too. However the method should be applied bearing in mind its restrictions due to the easy reduction of resazurin. As concerns the fragmented mycelia method, it is acceptable to be applied in cases of poor or no sporulating dermatophytes. The addition of resazurin sodium salt solution could be applied with safety to this method as well. The antifungal susceptibility testing of dermatophytes could serve as an important laboratory tool of great clinical importance. The available methods address to all types of dermatophytes permitting the laboratory to apply the most suitable and reliable method for each case.

## Data Availability Statement

The datasets generated for this study are available on request to the corresponding author.

## Author Contributions

A-MM and KS conducted the experiments, performed the statistical analysis, and wrote the manuscript. EZ contributed in the preparation of all the necessary material for the experiments. T-AV supervised the whole procedure, provided guidance, and edited the manuscript. All authors contributed to the article and approved the submitted version.

## Conflict of Interest

The authors declare that the research was conducted in the absence of any commercial or financial relationships that could be construed as a potential conflict of interest.
